# Utility of 18F-FDG PET/CT for diagnosing and staging solitary malignant tumors of the spleen

**DOI:** 10.3389/fmed.2025.1647670

**Published:** 2025-11-03

**Authors:** Mingyan Shao, Rong Xu, Sisi Fan, Wanling Qi

**Affiliations:** ^1^Department of Nuclear Medicine, Jiangxi Provincial People’s Hospital, The First Affiliated Hospital of Nanchang Medical College, Nanchang, China; ^2^Department of Pathology, Jiangxi Provincial People’s Hospital, The First Affiliated Hospital of Nanchang Medical College, Nanchang, China

**Keywords:** solitary malignant tumors of the spleen, spleen, diagnose, CT, 18F-FDG PET/CT

## Abstract

**Objective:**

To evaluate the diagnostic and staging value of 18F-FDG PET/CT in solitary malignant tumors of the spleen (SMTS).

**Methods:**

We retrospectively analyzed clinical data, 18F-FDG PET/CT and contrast-enhanced CT findings from 25 pathologically confirmed SMTS cases. The PET/CT characteristics including lesion distribution, accompanying signs, maximum standardized uptake value (SUVmax), and pathological types were analyzed, with comparison of diagnostic and staging efficacy between PET/CT and contrast-enhanced CT.

**Results:**

All 25 SMTS cases showed FDG uptake on PET/CT: mild uptake in 2 cases (SUVmax 2.4 and 2.3 respectively), moderate uptake in 8 cases (SUVmax 6.4 ± 3.5), and intense uptake in 15 cases (SUVmax 15.8 ± 4.2). The metabolic patterns included homogeneous uptake (13 cases), rim-like uptake (10 cases), and diffuse uptake (2 cases). CT morphology revealed round/oval lesions in 16 cases (64%, longest diameter 11.8 ± 4.5 cm), irregular lesions in 7 cases (28%, 17.6 ± 5.7 cm), and diffuse splenomegaly in 2 cases (8%, 18.4 ± 2.7 cm). Lesions demonstrated well-defined margins (4 cases) or ill-defined margins (21 cases), with central necrosis/cystic degeneration/hemorrhage (13 cases) or solid appearance (12 cases). Contrast-enhanced CT showed mild (7 cases, 28%), moderate (8 cases, 32%), and marked enhancement (9 cases, 36%). Pathological types included lymphoma (14 cases, mostly showing homogeneous hypermetabolism except 2 MALT lymphomas with hypometabolism), angiosarcoma (7 cases, all demonstrating rim-like uptake with with photopenia in the necrotic centers), other sarcomas (3 cases including 2 EBV-positive inflammatory follicular dendritic cell sarcomas with well-defined hypermetabolic lesions and 1 unclassified sarcoma with heterogeneous uptake), and solitary metastasis (1 case showing nodular hypermetabolism). PET/CT demonstrated significantly higher diagnostic sensitivity (84% vs. 68%, *p* = 0.012) and superior staging capability (28% vs. 12%, *p* = 0.027) compared to CT, the diagnostic sensitivity of PET/CT combined with enhanced CT was 96%.

**Conclusion:**

18F-FDG PET/CT provides significant diagnostic and staging value for SMTS, with SUVmax and metabolic patterns improving diagnostic accuracy. The combination of PET/CT and pathological biopsy is recommended for optimal evaluation.

## 1 Introduction

Solitary malignant tumor of the spleen (SMTS) represents a rare and heterogeneous entity, primarily comprising lymphoid and mesenchymal-origin neoplasms with an estimated annual incidence of approximately 0.1 per 100,000 population (1). The pathological spectrum is complex, including vascular-origin tumors (55%), lymphohematopoietic malignancies (30%), and mesenchymal tumors (15%) (2). Owing to the spleen’s deep anatomical location and the frequent absence of early symptoms, most SMTS are incidentally discovered at advanced stages, leading to considerable diagnostic challenges. Conventional imaging modalities, such as ultrasound, CT, and MRI, struggle to reliably differentiate SMTS from benign lesions like hemangiomas or hamartomas. While 18F-FDG PET/CT integrates metabolic and anatomical information—offering a potential solution for detecting occult disease and guiding biopsy—the existing evidence supporting its use in SMTS remains sparse and inconclusive. Prior reports, limited mostly to isolated case series, have suggested variable and often suboptimal sensitivity for PET/CT in characterizing splenic lesions, particularly for low-grade or early metastatic involvement (3–5). In this context, our retrospective analysis of 25 pathologically confirmed SMTS cases aims to address this critical knowledge gap. By systematically evaluating the clinical utility of 18F-FDG PET/CT, this study not only validates its role in diagnosis and staging but also provides novel, cohort-based evidence on its diagnostic performance, thereby offering clinicians a more robust foundation for therapeutic decision-making in this rare condition.

## 2 Materials and methods

### 2.1 Study population

We retrospectively analyzed 25 cases of solitary splenic tumors treated at our institution between April 2020 and April 2025.

Inclusion criteria were: (1) histopathological confirmation of solitary splenic malignancy; (2) completion of both 18F-FDG PET/CT and contrast-enhanced CT examinations prior to treatment; and (3) availability of diagnostic-quality imaging.

Exclusion criteria included: (1) history of other malignant tumors; (2) confirmed diagnosis of splenic metastatic tumors; and (3) previous splenic surgery or radiotherapy. Informed consent for the anonymous use of clinical data and imaging for publication purposes was provided by all patients. Given the retrospective nature of this study, the institutional review board waived the requirement for approval.

### 2.2 Imaging protocol

18F-FDG PET/CT examinations were performed using a Discovery-64CT scanner and MINItrace cyclotron (GE Healthcare). 18F-FDG was synthesized by a TRACERLab FX-FN synthesizer with radiochemical purity >96%. Patients fasted for ≥4 h prior to examination, with height, body weight, and blood glucose measured (maintained <10 mmol/L). Intravenous injection of 18F-FDG (5.55 MBq/Kg) was administered via antecubital vein, followed by 40–60 min rest in a quiet, dimly lit room. Patients consumed 800–1,000 mL water and voided before scanning. The protocol acquired 7 bed positions using CT first (120 kV, 20–200 mA) followed by PET (FORE-Iterative reconstruction, 50 cm × 50 cm FOV, 25-min emission scan, 4.25 mm axial interval, 3.75 mm slice thickness, 128 × 128 matrix). Scanning range extended from skull vertex to upper femoral third in supine position with arms raised. Image fusion and multiplanar reconstruction were performed on AW4.6 workstation. Contrast-enhanced CT used a 16-slice GE scanner (120 kV, 200 mA, 1.25 mm slice thickness, 5 mm interval) with automatic reconstruction of 1–1.5 mm thin slices (Lung algorithm, 500 mm × 500 mm FOV, 512 × 512 matrix). Iopromide (300 mgI/mL, Bayer AG) was injected at 1.5–2.0 mL/kg (2.5 mL/s flow rate) via power injector. All patients underwent standard preparatory protocols for contrast administration, including renal function screening (eGFR >45 mL/min/1.73m^2^), allergy assessment, and 4-h fasting prior to CT enhancement.

### 2.3 Image analysis

ROI-based semiquantitative analysis was performed on AW4.6 workstation. SUVmax was measured to reflect maximum radiotracer uptake, normalizing for dose, timing, and biodistribution variables. CT enhancement values, lesion size, morphology and density were measured using built-in tools. Based on splenic tumor FDG uptake, our research team categorized lesions into three grades according to SUVmax values in an institutional cohort: Mild uptake: SUVmax 0–2.5, Moderate uptake: SUVmax 2.5–10.0, Marked uptake: SUVmax >10.0. All PET/CT and CT images were independently reviewed by two board-certified radiologists (Wangling Qi and Mingyan Shao, with 8 and 18 years of experience, respectively) blinded to clinical data and patient allocation, with discordant cases resolved by panel consensus.

### 2.4 Statistical analysis

SPSS 26.0 was used for data analysis. Normality was verified using Shapiro–Wilk tests (*α* = 0.05) with visual histogram inspection. All variables satisfied normality criteria (*p* < 0.05). Where assumptions were violated, Welch’s *t*-test and Mann–Whitney U results were compared, with consistent findings. Normally distributed continuous variables were expressed as mean ± SD, with between-group comparisons using independent *t*-tests (statistical significance at *p* < 0.05).

## 3 Results

### 3.1 18F-FDG PET/CT findings

The baseline characteristics of the 25 SMTS cases are summarized in Table 1. All cases demonstrated FDG uptake on PET/CT, categorized as: mild uptake in 2 cases (SUVmax 2.4 and 2.3, respectively), moderate uptake in 8 cases (SUVmax 6.4 ± 3.5), and intense uptake in 15 cases (SUVmax 15.8 ± 4.2). The metabolic patterns included homogeneous FDG uptake (13 cases), peripheral rim-like FDG uptake (10 cases), and diffuse FDG uptake (2 cases). CT morphological features revealed round/oval lesions in 16 cases (64%; maximum tumor diameter 11.8 ± 4.5 cm), irregular lesions in 7 cases (28%; 17.6 ± 5.7 cm), and diffuse splenomegaly in 2 cases (8%; 18.4 ± 2.7 cm). Lesion margins were well-defined in 4 cases and ill-defined in 21 cases, with central necrosis/cystic degeneration/hemorrhage observed in 13 cases and solid appearance in 12 cases. Contrast-enhanced CT demonstrated mild enhancement (7 cases, 28%), moderate enhancement (8 cases, 32%), and marked enhancement (9 cases, 36%).

**Table 1:** Baseline characteristics of 25 cases with solitary malignant tumors of the spleen (*n* = 25).

Characteristics	Numerical value	Percentage
Age (years)	68.3 ± 12.5	–
Gender		
Male	13	52%
Female	12	48%
Clinical symptom		
Abdominal pain	11	44%
Persistent low-grade fever with fatigue	10	40%
Progressive emaciation	23	92%
Hypersplenism	12	48%
Tumor markers		
Elevated CA19-9 levels	13	52%
Elevated CEA levels	5	20%
Negative	12	48%
Tumor Size (cm)		
Round/Oval	11.8 ± 4.5	64%
Irregular	17.6 ± 5.7	28%
Diffuse	18.4 ± 2.7	8%
The pathological type		
Lymphadenoma	14	56%
Angiosarcoma	7	28%
Other sarcomas	3	12%
Metastatic neoplasm	1	4%
FDG uptake		
Mild (SUVmax) (0–2.5)	2	8%
Moderate (SUVmax) (2.5–10.0)	8	32%
Marked (SUVmax) (>10.0)	15	60%
CT Enhancement		
Mild (10–30 HU)	7	28%
Moderate (30–60 HU)	8	32%
Marked (>60 HU)	9	36%

**Figure 1:**
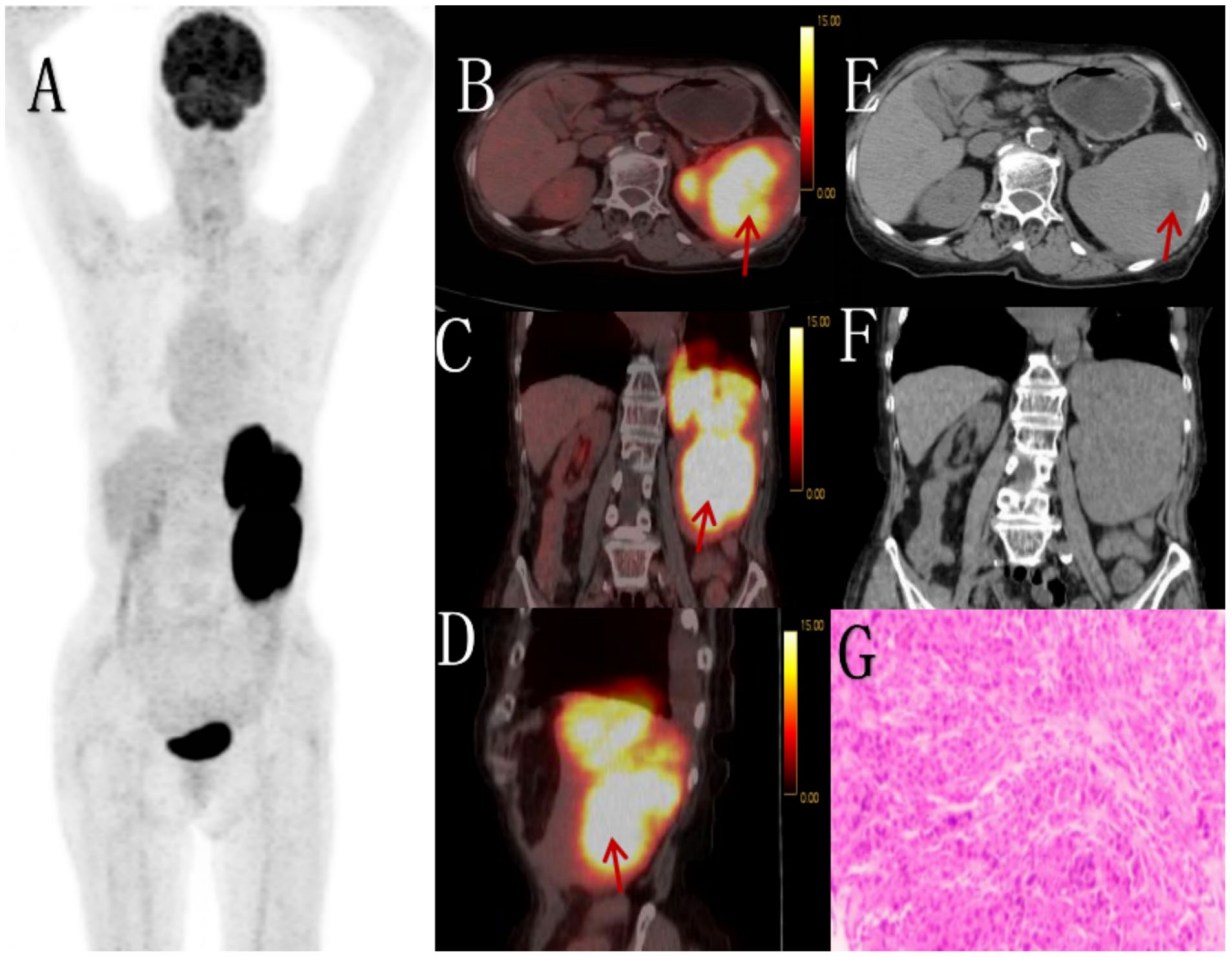
Female, 85 years old, diffuse large B-cell lymphoma. **(A)** whole body MIP, **(B)** axial fusion, **(C)** coronal fusion, **(D)** sagittal fusion, **(E)** axial CT, **(F)** coronal CT, **(G)** H-E × 200. 18F-FDG PET/CT demonstrated splenomegaly with an irregular soft tissue density mass (maximum cross-sectional dimension 17.7 × 8.5 cm) showing heterogeneous density and small hypodense necrotic areas. The lesion exhibited uniformly increased radiotracer uptake with a maximum standardized uptake value (SUVmax) of 14.4 (arrow, **B–D**). CT demonstrates an enlarged spleen with heterogeneous attenuation, showing multiple nodular and small patchy hypodense lesions. On contrast-enhanced scans, the spleen exhibits mild enhancement, while the necrotic areas show no significant enhancement **(E)**. PET/CT-guided splenic biopsy confirmed the diagnosis of diffuse large B-cell lymphoma, non-germinal center subtype **(G)**.

**Figure 2:**
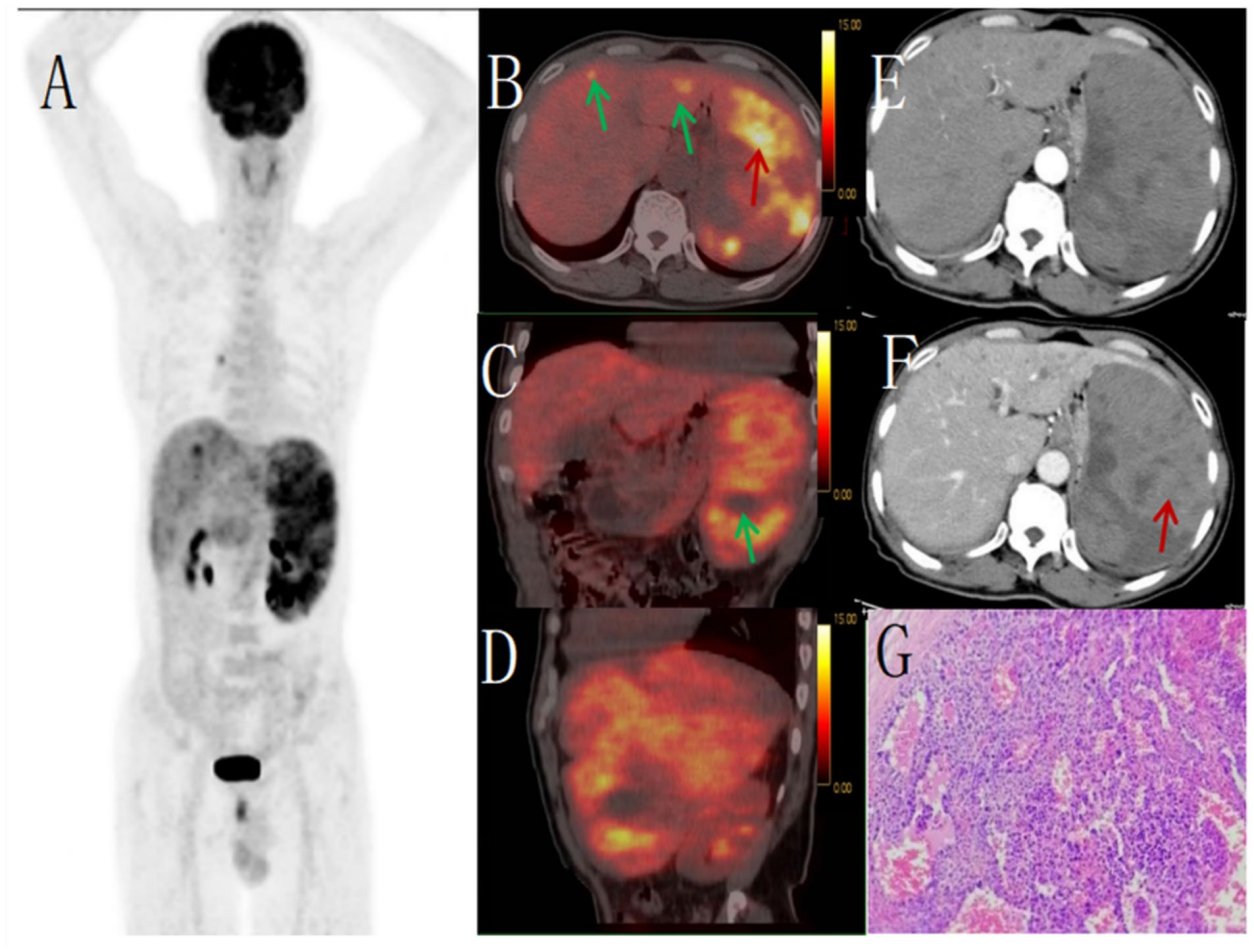
Male, 67 years old, primary splenic angiosarcoma. **(A)** whole body MIP, **(B)** axial fusion, **(C)** coronal fusion, **(D)** sagittal fusion, **(E)** enhanced CT-arterial phase, **(F)** enhanced CT-venous phase, **(G)** (H-E × 400). 18F-FDGPET/CT showed multiple nodular and massive lesions in the spleen with multiple necrotic areas, PET/CT images showed an abnormal increase in diffuse and uneven uptake of 18F-FDG in the spleen, with a SUVmax of 9.0 (red arrow, **B**) and no FDG uptake in some necrotic areas (green arrow, **C**). There were multiple nodular lesions in the liver, the level of FDG metabolism increased, the SUVmax was 5.6 (green arrow, **B**). Enhanced CT showed diffuse uneven mild enhancement of spleen, and the degree of enhancement increased with time (red arrow, **F**). Multiple nodular low-density shadows were seen in the liver, which were slightly enhanced by enhanced scan. PET/CT-guided splenic biopsy confirmed the diagnosis of primary splenic angiosarcoma **(G)**.

**Figure 3:**
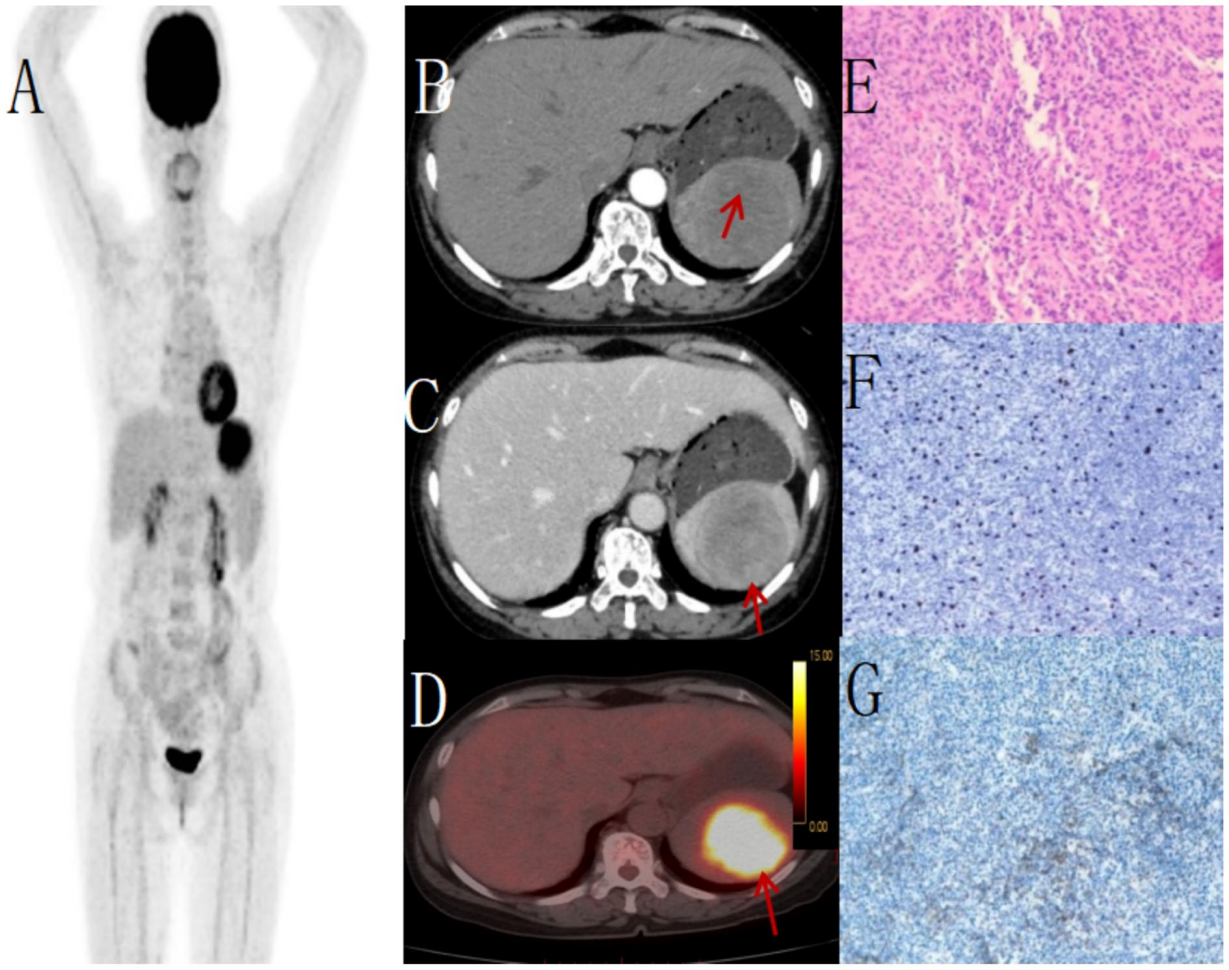
Female, 68 years old, EBV + IFDCS. **(A)** whole body MIP, **(B)** enhanced CT-arterial phasel, **(C)** enhanced CT-venous phase, **(D)** axial fusion, **(E)** H-E × 400, **(F)** Immunohistochemistry CD35, +, **(G)** EBER-by in-situ hybridization, +. 18F-FDGPET/CT showed circular low-density shadows in the spleen with clear boundaries, uneven internal density and low-density necrosis. FDG metabolism was significantly increased, showing uneven clumps of increased FDG metabolism, SUVmax was 7.8 (arrow, **D**). Non-contrast CT showed an enlarged spleen volume, a quasi-circular low-density mass with clear boundaries and necrosis in the spleen. Enhanced CT showed uneven delayed enhancement of the solid part, mild to moderate enhancement of the tumor in arterial stage, progressive enhancement in venous stage and delayed stage (arrow, **C**), no enhancement of necrosis, and mild pseudoenvelope enhancement (arrow, **B**). The tumor cells appeared oval and were arranged in bundles, nodules or small nests. The cytoplasm was eosinophilic, lightly stained, and the nuclear chromatin was fine, with small nucleolus and no mitotic image **(E)**.

**Figure 4:**
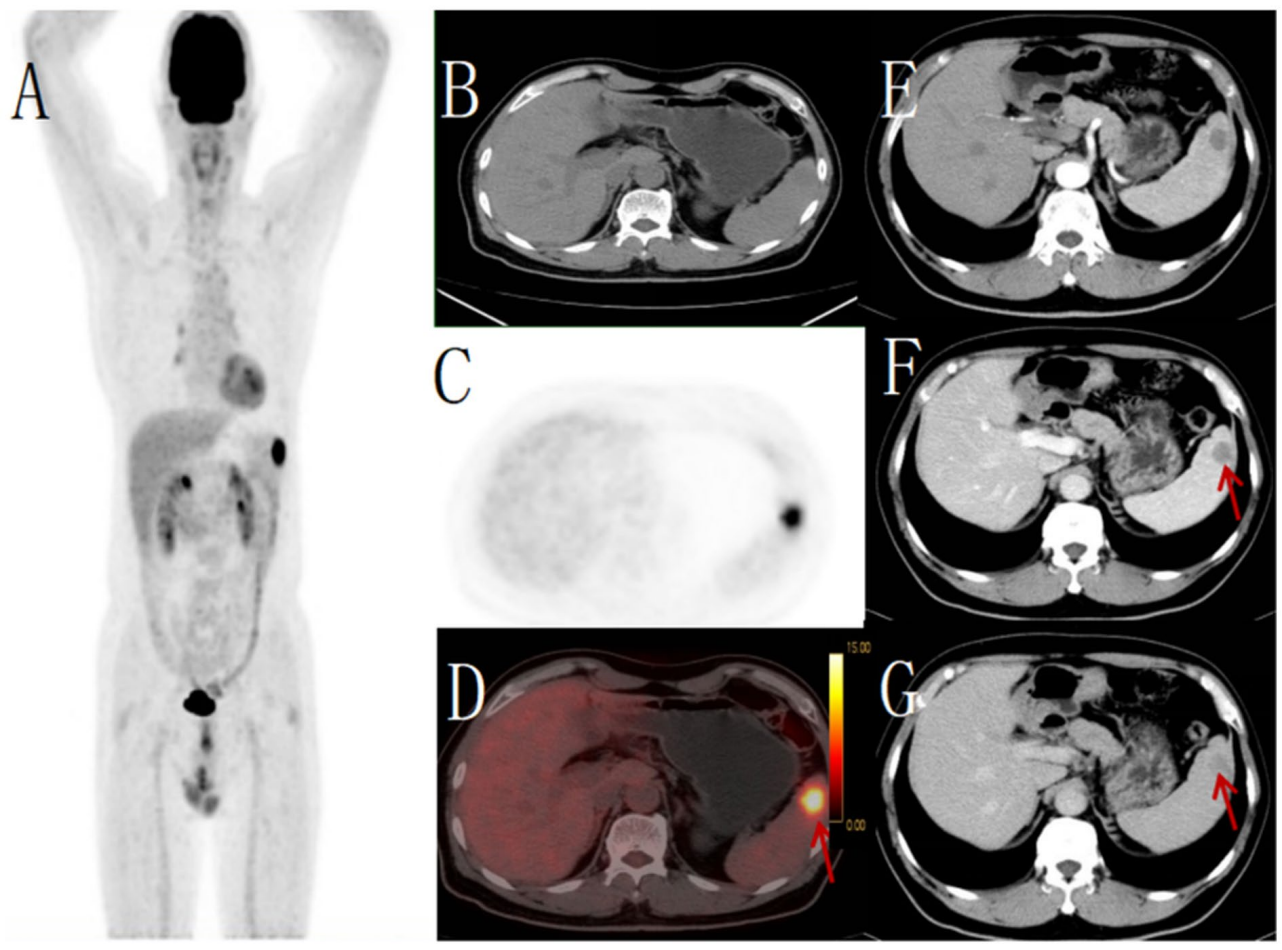
Male, 62 years old,metastatic tumor. **(A)** whole body MIP, **(B)** axial CT, **(C)** axial PET, **(D)** axial fusion, **(E)** enhanced CT-arterial phase, **(F)** enhanced CT-venous phase, **(G)** enhanced CT-delayed phase. 18F-FDG PET/CT revealed a round hypodense lesion (approximately 21 × 21 mm) with ill-defined margins in the upper pole of the spleen, demonstrating increased radiotracer uptake (SUVmax 11.5) (arrow, **D**). No other abnormal FDG-avid foci were detected elsewhere in the body. Contrast-enhanced CT showed a corresponding round slightly hypodense lesion within the splenic parenchyma, exhibiting mild peripheral enhancement (arrow, **F,G**). Pathological examination of the biopsy specimen confirmed the diagnosis of metastatic carcinoma.

Pathological classification of SMTS revealed the following characteristics: Among the lymphoma cases (*n* = 14), diffuse large B-cell lymphoma (*n* = 10) and splenic marginal zone B-cell lymphoma (*n* = 2) demonstrated homogeneous intense FDG uptake on 18F-FDG PET/CT, while the 2 cases of MALT lymphoma exhibited homogeneous low FDG uptake. Contrast-enhanced CT showed variable enhancement patterns (mild, moderate, or marked) with non-enhancing necrotic areas. All 7 angiosarcoma cases displayed characteristic peripheral rim-like FDG uptake with central photopenia on 18F-FDG PET/CT, accompanied by marked contrast enhancement with rapid wash-in/wash-out kinetics on CT. Of the other sarcomas (n = 3), EBV-positive inflammatory follicular dendritic cell sarcomas (*n* = 2) presented as well-circumscribed lesions with homogeneous hypermetabolism on PET/CT and heterogeneous delayed enhancement with non-enhancing necrotic/cystic areas on CT. The remaining unclassified sarcoma case (*n* = 1) showed heterogeneous FDG uptake with mild contrast enhancement. The solitary metastatic case demonstrated focal nodular hypermetabolism on PET/CT with mild peripheral enhancement on contrast-enhanced CT. Representative images are shown in Figures 1–4.

### 3.2 Comparison of diagnostic efficacy between PET/CT and contrast-enhanced CT for SMTS

Among 25 cases of solitary malignant splenic tumors (SMTS), preoperative PET/CT correctly identified 21 cases versus 17 by contrast-enhanced CT alone. Implementation of a combined PET/CT and contrast-enhanced CT approach increased diagnostic accuracy to 24 correct diagnoses. Statistical analysis confirmed significantly higher sensitivity with PET/CT compared to contrast-enhanced CT alone (*p* < 0.05), and further demonstrated superior sensitivity for the combined protocol relative to either individual modality, as detailed in Table 2. Specifically, PET/CT achieved a sensitivity of 92.8% (13/14) for primary splenic lymphoma, markedly superior to CT’s 78.6% (11/14). Additionally, PET/CT showed a sensitivity of 71.4% (5/7) for splenic angiosarcoma, outperforming CT’s 57.1% (4/7). Comparison of SUVmax in splenic lesions across different pathological subtypes (Lymphoma, Angiosarcoma, Other Sarcomas, Solitary Splenic Metastasis) was performed using one-way analysis of variance (ANOVA), which revealed no statistically significant difference among the groups. Using Spearman’s correlation analysis, we investigated the relationship between SUVmax on PET/CT and various parameters in cases of solitary splenic lymphoma and splenic angiosarcoma. In solitary splenic lymphoma, SUVmax showed a strong correlation with pathological subtypes: diffuse large B-cell lymphoma exhibited significantly higher SUVmax values compared to follicular lymphoma and marginal zone lymphoma. However, within the lymphoma group, SUVmax demonstrated no significant correlation with either the degree of contrast enhancement on CT or tumor lesion size. Conversely, in splenic angiosarcoma, SUVmax exhibited a significant positive correlation with the degree of CT contrast enhancement, but no significant correlation with tumor lesion size. Furthermore, in the two cases of Epstein–Barr virus-positive inflammatory follicular dendritic cell sarcoma (EBV + IFDCS), we observed that the case with higher CT contrast enhancement also had a higher SUVmax value compared to the case with lower enhancement.

**Table 2:** Comparison of preoperative PET/CT and contrast-enhanced CT diagnostic efficacy in 25 SMTS cases.

Method of inspection	Correct diagnosis of cases (cases)	Relevance ratio (%)	*p* value
PET/CT	21	84	0.012^a^
Enhancement CT	17	68	0.032^b^
Combine both imaging modalities	24	96	0.016^c^

a *p* value for PET/CT vs. Enhancement CT (McNemar test).

b *p* value for Enhancement CT vs. PET/CT (McNemar test).

c *p* value for Combined modalities vs. Enhancement CT (exact McNemar test).

### 3.3 The staging value of 18F-FDG PET/CT in SMTS

In this study of 25 cases of isolated splenic malignancies, PET/CT detected metastatic lesions in 7 patients, whereas CT identified only 3. The primary metastatic sites revealed by PET/CT included retroperitoneal lymph nodes, hepatic metastasis, pulmonary metastasis, and osseous metastasis, as detailed in Table 3. PET/CT led to stage migration (upstaging) in 28% (7/25) of splenic malignancies, significantly higher than the 12% (3/25) upstaging rate achieved by CT (*p* = 0.027). These findings demonstrate the critical role of PET/CT in tumor staging.

**Table 3:** Detection rates of metastatic lesions in 25 cases of splenic malignancies.

Pathological type	PET/CT Number of cases	Ratio (%)	CT Number of cases	Ratio (%)
Lymphadenoma				
Retroperitoneal lymph node	2	8	2	8
Osseous metastasis	1	4	0	0
No metastasis	11	44	12	48
Angiosarcoma				
Retroperitoneal lymph node metastasis	1	4	1	4
Hepatic metastases	1	4	0	0
Pulmonary metastasis	1	4	0	0
No metastasis	3	12	6	24
Other sarcomas				
Splenic hilar lymph node metastasis	1	4	0	0
No metastasis	2	8	3	12
Solitary metastatic tumor of spleen				
Other metastatic sites	0	0	0	0

## 4 Discussion

Solitary malignant tumor of the spleen (SMTS) is clinically rare, accounting for only 0.64% of all malignancies (6, 7). Their low incidence is attributed to splenic antitumor mechanisms, including: (1) mechanical filtration by the medullary system via CD8 + T cell-dependent immune clearance; (2) FasL-mediated apoptosis by sinusoidal endothelial cells; and (3) production of nonspecific antitumor substances (e.g., tuftsin, fibronectin, interferon-*γ*) coupled with abundant memory/helper T cells, collectively enabling tuftsin-dependent NK cell activation (8–10). Recent studies reveal that splenic macrophage subsets (e.g., CD206 + Tim4+) establish a “dual-barrier” mechanism through TGF-β1-dependent immunosuppression and FcγRIII-mediated tumor clearance (11–14). These findings provide a cytological basis for the rarity of isolated splenic tumors and suggest potential immunotherapeutic strategies targeting splenic macrophages.

Isolated splenic tumors often lack specific early manifestations. Clinical presentation depends on factors like splenomegaly severity, tumor location, and anatomical relationships. Common symptoms include left upper quadrant pain/discomfort, palpable masses, and anorexia. Systemic manifestations—such as persistent low-grade fever, progressive weight loss, fatigue, and hypersplenism—are more frequent in malignant cases (15, 16). In this study, solitary malignant splenic tumors presented with abdominal pain (44%), persistent low-grade fever with fatigue (40%), progressive emaciation (92%), and hypersplenism (48%). Mild CA199 elevation occurred in 52% of patients, while 48% showed tumor marker negativity. These findings indicate that clinical symptoms and tumor markers alone cannot reliably distinguish tumor nature. Accurate benign-versus-malignant differentiation remains clinically critical for treatment and prognosis. Traditional diagnosis relied on contrast-enhanced CT’s structural and vascular details, but for morphologically indeterminate lesions, 18F-FDG PET/CT provides definitive diagnosis by evaluating molecular characteristics, with reported sensitivity near 95% for Solitary malignant tumor of the spleen (17).

In this study, our systematic analysis of 25 pathologically confirmed cases demonstrates the pivotal value of 18F-FDG PET/CT. It achieved an 84% diagnostic accuracy for isolated splenic malignancies, which increased to 96% when combined with contrast-enhanced CT. All 25 cases of solitary splenic malignancies demonstrated increased FDG uptake, including 14 lymphomas, 7 angiosarcomas, 3 other sarcomas, and 1 isolated metastatic tumor. PET/CT showed a sensitivity of 92.8% (13/14) for primary splenic lymphoma, significantly higher than CT’s 78.6% (11/14). For splenic angiosarcoma, the sensitivity reached 71.4% (5/7), superior to CT’s 57.1% (4/7). Notably, we observed that splenic lymphomas (particularly DLBCL) exhibited homogeneous hypermetabolism (SUVmax 10.2 ± 3.5), Pathologically, FDG uptake intensity reflected lymphoma aggressiveness, with SUVmax values showing clear correlation with histological subtypes - the 2 MALT lymphomas in our series demonstrated low SUVmax (2.1–3.4), consistent with their indolent behavior, while other lymphomas showed SUVmax>5, indicating aggressive biology. In this study, SUVmax values showed no significant correlation with the degree of CT enhancement or tumor lesion size in splenic lymphomas. However, a clear correlation was observed with lymphoma subtypes, suggesting that tumor activity in splenic lymphoma may be associated with cellular immunohistochemical expression profiles. Diffuse large B-cell lymphoma (DLBCL) typically exhibits high Ki-67 expression (>80%), indicating high proliferative activity (18, 19), while MALT lymphomas generally demonstrate lower Ki-67 levels, corresponding to reduced tumor proliferation and aggressiveness. Angiosarcomas consistently displayed a characteristic “rim-like hypermetabolism with central necrosis” pattern on PET/CT, with markedly elevated FDG uptake (SUVmax 11.8 ± 4.2). Notably, SUVmax in splenic angiosarcomas correlated with the degree of CT enhancement, where greater enhancement was associated with higher SUVmax values. This elevated metabolic activity may be attributed to the tumor’s angiogenic properties, such as high expression of CD31 and CD34, explaining the observed hypermetabolic phenomenon (20–22). Other sarcomas, such as EBV-positive inflammatory follicular dendritic cell sarcoma, showed intense inflammatory infiltrates (lymphocytes, plasma cells, histiocytes, multinucleated giant cells, and eosinophils) with granuloma formation and collagen proliferation, accounting for their high metabolic activity (23). These overlapping metabolic features among different splenic malignancies present significant diagnostic challenges in clinical practice.

PET/CT demonstrates significant value in staging evaluation of splenic malignancies due to its unique whole-body metabolic-anatomical imaging capability (5). Critically, our study confirms PET/CT’s superior staging accuracy over CT alone, evidenced by stage migration (upstaging) in 28% of cases (7/25). This superiority was primarily driven by detection of occult metastases in lymph nodes (4 cases), liver (1 case), lung (1 case), and bone (1 case). Most significantly, PET/CT directly altered clinical management in 5 cases: systemic therapy was initiated instead of local treatment in 4 patients with CT-diagnosed “isolated splenic lesions” after PET/CT revealed distant metastases. Unnecessary splenectomy was avoided in 1 patient with PET/CT-detected bone metastasis. This staging advantage is mechanistically explained by PET/CT’s sensitivity to early metabolic changes, particularly for bone metastases where small/miliary lesions often evade CT detection due to absent structural changes. By integrating metabolic and anatomical data, PET/CT not only improves occult metastasis detection but serves as the gold-standard pre-therapeutic staging tool. Regarding biopsy guidance, PET/CT-targeted sampling of SUVmax-hotspots increased diagnostic yield to 92% (vs. 68% for conventional CT-guidance) (24, 25), as demonstrated in one angiosarcoma case where PET/CT achieved diagnosis after CT-guided biopsy failed. Through precise differentiation of viable tumor from necrosis, PET/CT significantly optimizes biopsy precision and therapeutic decision-making (26).

The diagnostic challenges of PET/CT observed in our cohort stem primarily from overlapping metabolic patterns between benign and malignant splenic conditions. While physiological splenic FDG uptake is typically mild (SUVmax <2.5) (27, 28), we encountered cases where infection (e.g., suspected splenic tuberculosis) or inflammatory states mimicked malignancy through focal hypermetabolism. Conversely, certain malignancies in our series—particularly low-grade lymphomas and early angiosarcomas—exhibited only mild metabolic activity, overlapping with benign physiology, consistent with literature reports (29–31). This metabolic ambiguity was evident in 3 cases where initial PET/CT interpretation required modification after integrating clinical history and laboratory results. For instance, one patient with autoimmune disease-related splenomegaly showed moderate FDG uptake (SUVmax 5.1) initially concerning for lymphoma, but normalized after immunosuppressive therapy. These observations highlight a key limitation: SUVmax thresholds alone cannot reliably distinguish malignancy in the spleen. To mitigate this, our protocol mandated comprehensive assessment including: Clinical context (e.g., fever, immune status), Laboratory markers (CRP, LDH, blood cultures), Contrast-enhanced CT characteristics. Delayed PET/CT imaging provides additional diagnostic value by assessing metabolic patterns (32), as demonstrated in our study where feasible implementation (retention index >10%) supported malignancy diagnosis in two upstaged cases. Despite these measures, PET/CT’s inherent limitations – including spatial resolution constraints (potentially missing subcentimeter lesions) and nonspecific metabolic overlap–necessitate histopathological confirmation for definitive diagnosis. This study has several limitations inherent to its design. As a series of 25 cases, the small sample size cannot encompass all diagnostic scenarios, as illustrated by the absence of hypometabolic metastases in our cohort. This reinforces the paramount importance of integrated clinical-radiologic-pathologic correlation. Furthermore, the retrospective, single-center nature limits the generalizability of our findings. Finally, the absence of a control group with benign conditions precludes a meaningful assessment of the model’s specificity, which is a crucial metric for its potential diagnostic utility.

In conclusion, 18F-FDG PET/CT demonstrates significant diagnostic utility in solitary splenic malignancies, where quantitative SUVmax parameters and distinct metabolic patterns substantially enhance diagnostic performance. Particularly, PET/CT exhibits remarkable clinical value in precise staging of isolated malignant splenic tumors. We therefore recommend the combined application of PET/CT imaging and pathological biopsy to maximize diagnostic confidence in clinical practice.

## Data Availability

The datasets presented in this study can be found in online repositories. The names of the repository/repositories and accession number(s) can be found in the article/supplementary material.
